# Glutathione Transferases Responses Induced by Microcystin-LR in the Gills and Hepatopancreas of the Clam *Venerupis philippinarum*

**DOI:** 10.3390/toxins7062096

**Published:** 2015-06-09

**Authors:** Mariana Carneiro, Bruno Reis, Joana Azevedo, Alexandre Campos, Hugo Osório, Vítor Vasconcelos, José Carlos Martins

**Affiliations:** 1 CIIMAR/CIMAR—Interdisciplinary Centre of Marine and Environmental Research, University of Porto, Rua dos Bragas 289, Porto 4050-123, Portugal; E-Mails: marianarcarneiro@gmail.com (M.C.); brunobuendia@gmail.com (B.R.); joana_passo@hotmail.com (J.A.); acampos@ciimar.up.pt (A.C.); vmvascon@fc.up.pt (V.V.); 2 IPATIMUP—Institute of Molecular Pathology and Immunology of the University of Porto, Porto 4200-465, Portugal; E-Mail: hosorio@ipatimup.pt; 3 Instituto de Investigação e Inovação em Saúde, University of Porto, Porto 4200-135, Portugal; 4 Faculty of Medicine, University of Porto, Porto 4200-319, Portugal; 5 Department of Biology, Faculty of Sciences, Porto University, Rua do Campo Alegre, Porto 4069-007, Portugal

**Keywords:** microcystins, glutathione transferases, enzyme activity, proteomics, real-time PCR, *V. philippinarum*, detoxification, biomarker

## Abstract

A multi-method approach was employed to compare the responses of Glutatione Transferases (GSTs) in the gills and hepatopancreas of *Venerupis philippinarum* to microcystins (MCs) toxicity. In this way, using the cytosolic fraction, the enzymatic activity of GSTs, superoxide dismutase (SOD), serine/threonine protein phosphatases (PPP2) along with the gene expression levels of four GST isoforms (pi, mu, sigma1, sigma2) were investigated in both organs of the clams exposed for 24 h to 10, 50 and 100 μg L^−1^ of MC-LR. Cytosolic GSTs (cGSTs) from both organs of the high dose exposed clams were purified by glutathione-agarose affinity chromatography, characterized kinetically and the changes in the expression of cGSTs of the gills identified using a proteomic approach. MC-LR caused an increase in GST enzyme activity, involved in conjugation reactions, in both gills and hepatopancreas (100 μg L^−1^ exposure). SOD activity, an indicator of oxidative stress, showed significantly elevated levels in the hepatopancreas only (50 and 100 μg L^−1^ exposure). No significant changes were found in PPP2 activity, the main target of MCs, for both organs. Transcription responses revealed an up-regulation of sigma2 in the hepatopancreas at the high dose, but no significant changes were detected in the gills. Kinetic analysis evidenced differences between gills of exposed and non-exposed extracts. Using proteomics, qualitative and quantitative differences were found between the basal and inducible cGSTs. Overall, results suggest a distinct role of GST system in counteracting MCs toxicity between the gills and the hepatopancreas of *V. philippinarum*, revealing different roles between GST isoforms within and among both organs.

## 1. Introduction

Cyanobacteria are a well-known source of environmental contamination and water-related concerns in the industrial sector, with worldwide occurrence records [1]. *Microcystis aeruginosa*, a common microcystin (MC) producer, is an example of such that can form extensive blooms in freshwater and estuarine habitats [2]. There are numerous accounts of these toxins’ presence in water bodies around the world. Among the several toxin congeners, the most commonly produced cyanotoxin is microcystin-LR (MC-LR) [3,4]. Although this toxin is considered a public health issue mainly in freshwater, MC-LR has already been found in marine habitats. For instance, MC-LR has been identified in mussels from different regions such as the Northeastern Pacific, European and eastern Canadian coasts [5]. In addition, in Monterey Bay National Marine Sanctuary (North America), MC-contaminated marine bivalves were considered as the most likely cause for the death of sea otters through tropic transfer [2]. So far, such occurrences can have two explanations, they can occur due to outflows of MC-contaminated freshwater to the sea or due to invading freshwater species that are able to produce these toxins [5]. Although it has been referred to, some doubt has arisen regarding the capability of marine prokaryotes to produce this toxin. As such, to our knowledge, documented cases of MC-LR found in marine environments have been linked to one of the two explanations given previously.

MC-LR is a hepatotoxin known to inhibit serine/threonine-specific phosphatases PPP1, 2, 4, 5 and 6 [6,7,8]. Such inhibitions increase protein phosphorylation causing cytotoxic effects and deregulation of cell division, which can result in a tumor-promoting activity [9,10,11]. It is also known that this toxin is able to induce oxidative damage and the disruption of osmoregulation [12,13,14,15,16,17,18]. Glutathione transferases (GSTs) constitute a defense mechanism against several xenobiotics including MC-LR. GSTs are a multigene family of enzymes (isoforms), majorly represented in the cytosol, responsible for phase II biotransformation processes [19]. GSTs isoforms are non-equally expressed in different species and in different tissues within the same species where they perform catalytic and non-catalytic functions [20]. They are categorized according to their sequence homology, immunological cross-reactivity and ability to catalyze the nucleophilic addition of the tripeptide glutathione (GSH) thiol group to a broad range of xenobiotics with electrophilic functional groups [20,21]. The resulting reaction between MC-LR’s methylene group at the Mdha site with the –SH group of GSHs or cysteine residues, reveals a neutralized electrophilic site and a more water-soluble molecule [22,23,24]. GSTs can also serve as peroxidases and isomerases, protecting cells against oxidative damage [25]. The formation of MC-LR-GSH conjugates was demonstrated *in vitro* in several aquatic organisms including bivalves, and is suggested as the first step in the detoxification of MCs in these organisms [24,26,27].

Although the information regarding uptake and toxicity mechanisms of this cyclic hepatotoxin in mammals is somewhat extensive, the same cannot be said for aquatic animals [28]. Aquatic organisms are especially subjected to a more direct and frequent contact with MCs. In these organisms, the detoxification process is a biological adaptation of fundamental importance that may influence their ability to survive when cyanobacterial blooms occur. Being a sessile, filter feeding and lower trophic level class organism, bivalves are one of the most threatened groups by cyanotoxins [29]. As such, bivalves are suitable organisms for biomonitoring, and are also a good target to assess the risk of MC-LR exposure to consumers through bioaccumulation [29,30,31,32]. *V. philippinarum* is an invasive clam species from the Indo-Pacific region with economic importance in several European countries. This species competes directly with the European native clam (*Ruditapes decussatus*) for the same habitats and resources. After the introduction of the invasive clam in Europe, a massive decrease of the native one has been registered in result of its higher growth rates and higher resistance to physical stress and pathogens [33,34]. Therefore, it is important to understand the different defense mechanisms surrounding them in contrast to similar clams. Bivalves are capable of withstanding baseline levels of pollution, showing relative insensitivity to toxicants compared to other aquatic organisms. Being included in the human diet, the contamination and persistence of MCs in bivalves can pose a serious threat to public health and damage to the fishing industry. Rita *et al.* [35] established a direct relationship between environmental pollution and food safety, caused by *Mytilus galloprovincialis* contamination with MCs from freshwater toxic blooms. These facts highlight the potential role of detoxification enzymes such as GSTs in bivalve resistance to these toxicants. Although, many novel GST classes have been identified and classified from non-mammalian organisms, information on bivalve GSTs is still scarce. However, some work has already connected the involvement of the GST detoxification enzyme system to the molecular response to MCs exposure in bivalves [24,36,37]. MC-LR-induced toxicity depends on the levels and duration of internal exposure, determined by the balance between absorption, detoxification, and excretion [38]. The diversity of GST isoforms and their catalytic promiscuity can be seen as an advantage when in chemical stress. In this way, the identification of specific isoforms involved, individually or not, in the biotransformation and biodegradation of xenobiotics is essential. In this sense, in bivalves, two organs stand out in detoxification studies: the gills, for being in direct contact with the water, and the hepatopancreas where the biotransformation of xenobiotics mainly occurs.

In this light, the aims of this study were to provide information on the molecular behavior of *V. philippinarum* upon exposure to different concentrations of MC-LR, on the organism’s tissue specificities (gills and hepatopancreas) regarding the detoxification role of MC-LR via the GST system, as well as which GST isoforms are more prone to act in the organism’s defense against MCs toxicity. In order to do so, enzymatic responses (GST, SOD and PPP2) and gene expression levels of four GST isoforms (pi, mu, sigma1 and sigma2) were assessed in the gills and hepatopancreas of *V. philippinarum* in an exposure assay using 10, 50 and 100 μg L^−1^ of purified MC-LR (2.5, 12.5 and 25 μg of MC-LR available per clam) [36,39]. These concentrations fall in the range of values found in natural waters, that can go from trace concentrations up to 1800 μg L^−1^ or higher, immediately after the collapse of a highly toxic bloom [40]. Afterwards, cytosolic GSTs from both organs of the high dose and control groups were purified and the extracts characterized kinetically. Based on these results, the changes in the expression of the gills cGSTs were scrutinized through bidimensional electrophoresis. This multi-approach may help to contribute to the knowledge concerning the molecular mechanisms of MC induced toxicity in bivalves.

## 2. Results

### 2.1. Enzyme Activity

#### 2.1.1. GST Activity

**Figure 1:**
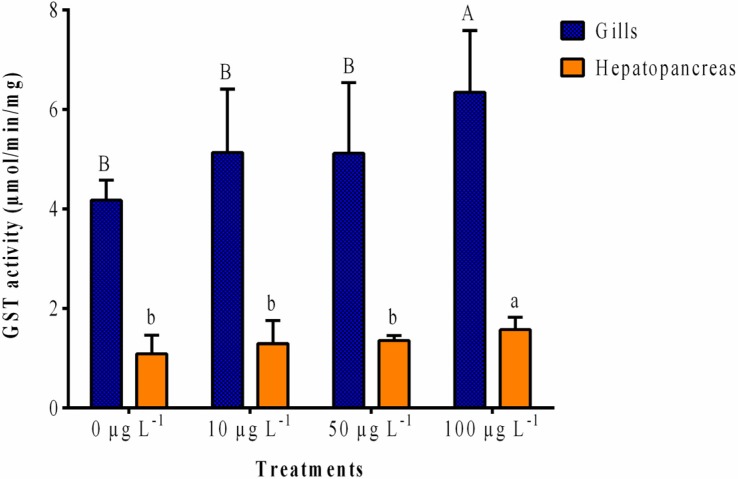
Glutatione transferases (GST) activity expressed as μmol of 1-chloro-2,4-dinitrobenzene glutathione (CDNB-GSH) conjugate per min per mg of protein in the gills and hepatopancreas of *V. philippinarum* exposed to three different concentrations of purified microcystin-LR (MC-LR). Data are expressed as mean ± SD (*n* = 3). Capital letters are depicted for the gills and lowercase letters for the hepatopancreas. Treatments that do not share a letter are significantly different (*p* < 0.05).

The wide-ranging substrate used, 1-chloro-2,4-dinitrobenzene (CDNB), catalyzes most of the known GST isoforms [41]. In our work, GST activity levels were consistently lower in the hepatopancreas than in the gills (more than three-fold). GST activity from both organs showed an increase trend with increasing MC-LR levels (Figure 1). However, a significant (*p* < 0.05) increase (1.5-fold for both organs) in relation to control was detected for the high dose exposed group (100 μg L^−1^) in the gills and hepatopancreas.

#### 2.1.2. SOD Activity

**Figure 2:**
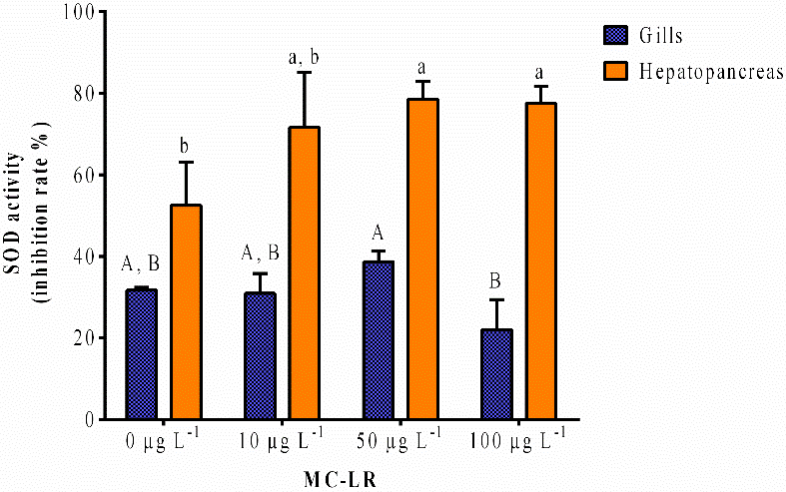
Superoxide dismutase (SOD) and SOD-like activities expressed as percentage of inhibition rate of the gills and hepatopancreas of *V. philippinarum* exposed to three different concentrations of purified MC-LR and the respective controls. Data are expressed as mean ± SD (*n* = 3). Capital letters are depicted for the gills and lowercase letters for the hepatopancreas. Treatments that do not share a letter are significantly different (*p* < 0.05).

SOD enzymes are part of one of the organism defenses against reactive oxygen species (ROS). In this study it is possible to see a clear increase of SOD activity in the hepatopancreas alongside the growth of MC-LR concentration, with statistical differences between the 50 μg L^−1^ and 100 μg L^−1^ exposures and the control (Figure 2). However, in the gills, the results were not so linear, with a slight increase in activity on the 10 μg L^−1^ and 50 μg L^−1^ exposed group, followed by a significant decline in the 100 μg L^−1^ exposure.

The results in the gills do not disclose changes relative to the control, nor is a clear trend perceived among different exposures of MC-LR. Basal SOD activity in the hepatopancreas of *V. philippinarum* revealed to be significantly (*p* < 0.05) higher than in the gills (1.7-fold).

#### 2.1.3. PPP2 Activity

**Figure 3:**
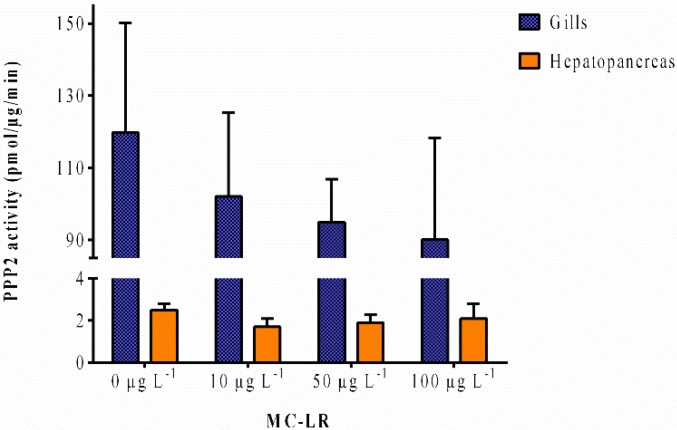
Serine/threonine protein phosphatases (PPP2) activity measured in the gills and hepatopancreas of *V. philippinarum* exposed to purified MC-LR expressed as pmol per μg of protein per minute. Data are expressed as mean ± SD (*n* = 3).

Known to be a powerful inhibitor of PPP2 catalytic subunit leading to a cellular hyperphosphorylation state and being a potent liver tumor promoter as well, MC-LR can cause severe damages to the organism [42]. The PPP2 activity is significantly (*p* < 0.05) higher (47-fold) in the gills than in the hepatopancreas (Figure 3). Although there are no significant differences between exposures, there is a decrease in PO_4_ with the rise in the exposure’s intensity in the gills, while in the hepatopancreas there is no apparent change in response to the MC-LR exposure. Nonetheless, it is noteworthy the difference in PPP2 inhibition between both organs.

### 2.2. Gene Expression

**Figure 4:**
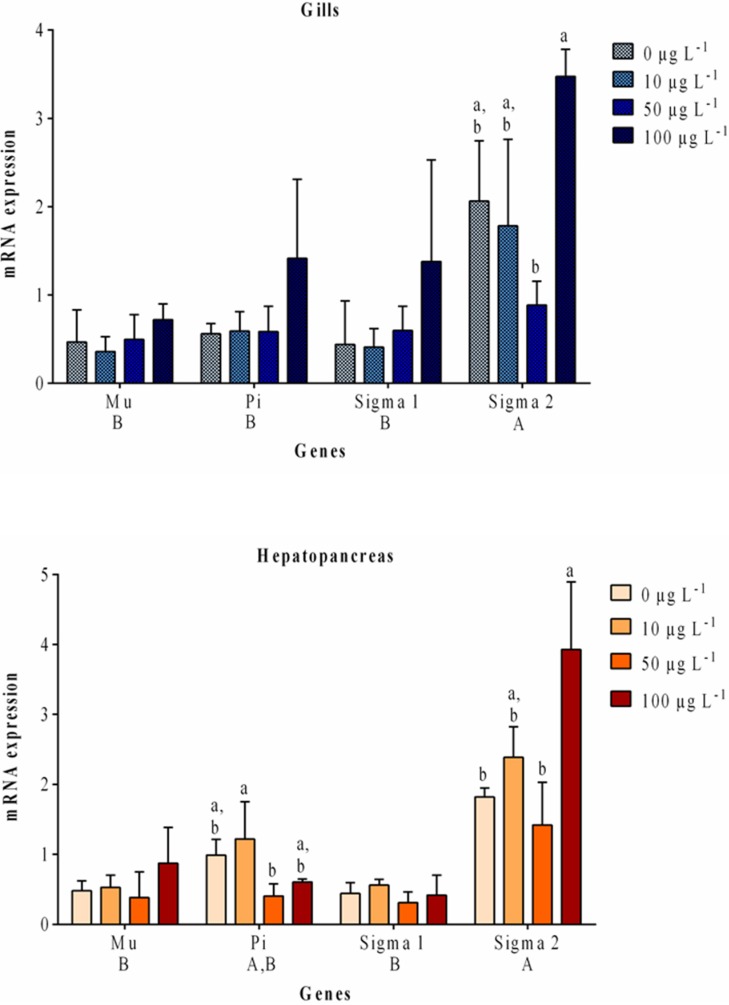
Gill and hepatopancreas temporal changes of GSTs transcripts of *V. philippinarum* after exposure to three different concentrations of purified MC-LR and the respective controls. Data are expressed as mean ± SD (*n* = 3). Differences between treatments are considered among each gene and are represented as lowercase letters. Differences between genes are represented as capital letters depicted below each gene’s name. Treatments and genes that do not share a letter are significantly different (*p* < 0.05).

In this study, the relative changes of the mRNA abundance of mu, pi, sigma1 and sigma2 GST isoforms were evaluated in both gills and hepatopancreas of *V. philippinarum* (Figure 4). In the gills, no significant changes were found for the transcription of all GST isoforms between the treatments and the control group. However, almost all GST isoforms (mu, pi, sigma1) in this organ presented a similar transcription trend in relation to the control group. Such was characterized by no changes for the 10 and 50 μg L^−1^ exposures and a slight increase for the highest exposure group, which in the case of pi and sigma1 isoforms is superior to 2.5-fold. On the other hand, the transcription of GST sigma2 decreased in relation to the control group upon the 10 and 50 μg L^−1^ MC-LR exposure (1.2- and 2.3-fold, respectively). Still, and in the same way as for the other GST isoforms, sigma2 transcription also increased after 100 μg L^−1^ treatment (1.7-fold). For sigma2 GST isoform, a significant change was found between the 50 and 100 μg L^−1^ treatment with MC-LR (*p* < 0.05). Our results indicate that among the four GST isoforms, sigma2 is the most abundantly expressed in the gills (*p* < 0.05) in non-exposed clams. In the hepatopancreas, the transcription of GST sigma2 significantly (*p* < 0.05) increased (2.2-fold) in relation to control for the highest exposure group (100 μg L^−1^). Similarly to the gills, this GST isoform also presents the same significant change between the 50 and 100 μg L^−1^ MC-LR exposure (*p* < 0.05). In this organ, no significant changes were found for the transcription of mu, pi and sigma1 GST isoforms between the treatments and the control groups. Nonetheless, a slight increase in transcription of the high dose group can be perceived (1.8-fold) for the mu GST isoform when compared to the control. In addition, a decrease of pi GST transcription in relation to control is perceived upon the 50 (2.4-fold) and 100 (1.6-fold) μg L^−1^ exposure. In fact, for this last isoform, a significant decrease was found between the 10 and 50 μg L^−1^ treatment (*p* < 0.05). Our data also shows that in gills no significant changes were found for the transcription of all the tested GST isoforms, although a noticeable increase of the transcription levels is found for all genes in the high dose group, with special relevance for pi and sigma1 GST isoforms. In the hepatopancreas, the transcripts of sigma2 were also dominantly expressed in relation to mu and sigma1 isoforms (*p* < 0.05) in non-exposed clams.

### 2.3. Enzyme Kinetics

**Figure 5:**
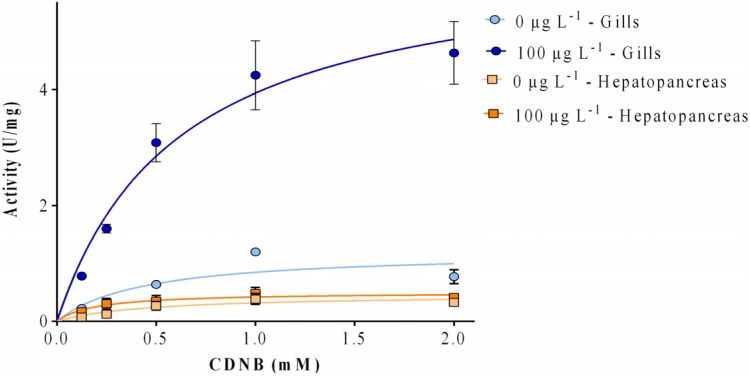
Michaelis-Menten representation of the effect of CDNB concentration on the GST activities of the gills and hepatopancreas of *V. philippinaru*m exposed to 100 μg L^−1^ and non-exposed animals, using the purified fraction. The regression coefficients (*R*) for goodness of fit for each samples are as follows: for the hepatopancreas 0.727 and 0.636 for the control and the exposed animals, respectively, and for the gills 0.666 and 0.926 for the control and the exposed animals, respectively.

Enzymatic analysis was performed by measuring the GST activity with 1 mM of GSH and varying CDNB substrate concentrations (0.125–2 mM). In Figure 5, the Michaelis-Menten representation of the kinetics of GST activity in the gills and hepatopancreas of *V. philippinarum* exposed to 100 μg L^−1^ of MC-LR and of non-exposed animals is shown. All samples demonstrated a classic Michaelis-Menten enzyme saturation curve using CDNB. The maximum activity can be observed at 2 mM CDNB in the exposed gills sample.

**Figure 6:**
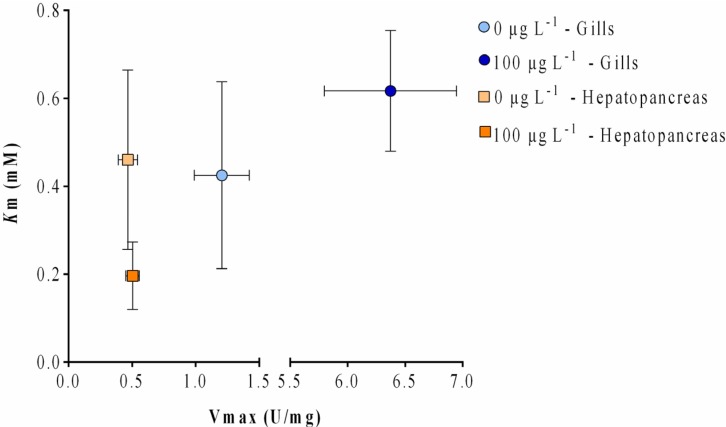
Comparison between apparent *K*_m_ and *V*_max_ values exported from the Michaelis-Menten fitted curve for the purified fractions of gills and hepatopancreas of *V. philippinarum* exposed to 100 μg L^−1^ and non-exposed animals using CDNB. Data is expressed as the mean of *n* = 1 for all samples.

The kinetic analysis in both organs shows that apparent *K*_m_ values decreased in the exposed hepatopancreas relatively to control, and increased in the exposed gills also in relation to its control, both without significance. On the other hand, apparent *V*_max_ values increased in animals exposed to 100 μg L^−1^ of MC-LR relatively to the respective controls, although only the gills revealed a significant change (*p* < 0.05) (Figure 6).

2.4. 2DE

**Figure 7:**
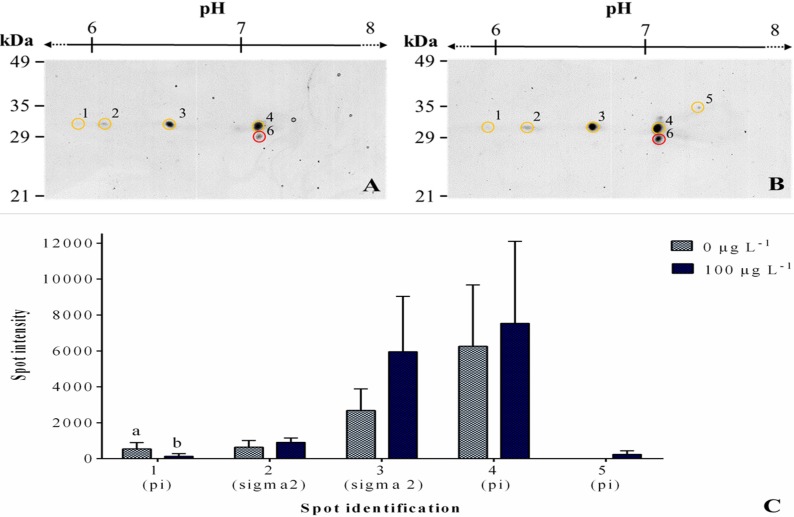
Cropped two-dimensional gel electrophoresis profile of *V. philippinarum* gills ((**A**) control group, (**B**) exposed to 100 μg L^−1^ of MC-LR group) stained with colloidal Coomassie Blue. Proteins identified as GSTs by MALDI-TOF/TOF mass spectrometry are delimited on these representative gels by a yellow circle, and non-GSTs with a red circle with the respective reference number on its right. Protein expressions of both treatments are depicted below (**C**). Intensity of spots of both treatments. Data are expressed as mean ± SD (*n* = 3). Treatments of spots that do not share a letter are significantly different (*p* < 0.05).

The affinity-purified cGST fractions of *V. philippinarum* gills exposed to 100 μg L^−1^ of MC-LR and non-exposed were analyzed by 2DE for the proteomic profiling of basal and inducible (expressed in response to different conditions) cGSTs mixtures. Protein profiles were reproducible among replicate gels from the same group (the control and the group exposed to 100 μg L^−1^). Cytosolic GSTs are, as a general rule, biologically active as dimers of subunits of 23–30 kDa [25]. Protein spots were resolved with molecular weights ranging between ~27–35 kDa and with an isoelectric point (pI) range of ~5.5–7.5 for both groups (Figure 7A,B). An average of five and six spots were resolved in the control and exposed groups, respectively. Analysis across protein profiles of the two experimental groups (control *vs.* exposed) using PD-Quest software revealed a quantitative as well as a qualitative difference in the abundance of specific proteins (Figure 7C). The quantitative change was observed for spot 1, which was four times under-expressed after exposure. The qualitative change was detected for spot 5, which was not expressed previously to the exposure. Comparison between treatments led to an overexpression of proteins, with the exception of spot 1. Afterwards, proteins that fitted the parameters (described in the methods section) were excised and selected for MALDI-TOF/TOF (Matrix-assisted laser desorption/ionization-time of flight/time of flight) analysis. The results for spot identifications are depicted in Table 1.

**Table 1:** Identification of the affinity chromatography purified in gel proteins using MALDI-TOF/TOF (Matrix-assisted laser desorption/ionization-time of flight/time of flight) and Mascot software. The database used was OrganismSpecie, with *V. philippinarum* as the organism, and with a confidence interval of 95% (*p* < 0.05).

Spot Number	Nominal Mass (Da)	Calculated pI	Putative Identification	Accession	Protein Score	Protein Sequence Coverage (%)	Ratio to Control
1	24023	7.67	GST pi-class	B9VAW9	333	49	−4.1
2	24516	6.64	GST sigma2-class	G9HSP3	815	50	1.4
3	24516	6.64	GST sigma2-class	G9HSP3	1460	85	2.2
4	24023	7.67	GST pi-class	B9VAW9	1090	67	1.2
5	24023	7.67	GST pi-class	B9VAW9	420	52	-

In relation to the purified cytosolic GSTs, nominal mass, calculated from integer atomic weights, was used due to the low molecular weight samples. However, nominal mass brings cumulative errors by approximating atomic weights with integers. Adding to this, protein ladders in 2DE gels can also contribute with a small error due to a more prone distortion of the gel mesh close to the gel borders. Both can explain the deviation of the molecular mass between the gel and the one provided through MASCOT software. However, according to the confidence interval and protein score, protein identification can be considered accurate. Of the six spots analyzed, five were identified as cGSTs subunits, with molecular masses between 24.023 and 24.516 kDa and isoelectric points (pI) values between 6.64 and 7.67. Spots 1, 4 and 5 were identified as putative pi-class GST subunits (B9VAW9) and spots 2 and 3 as a putative sigma2-class GST subunits (G9HSP3). A non-GST protein eluted from the glutathione-agarose affinity matrix was also identified (spot 6) as a histone H1 (accession: B3FEA3; result not shown).

## 3. Discussion

After 24 h exposure to three different concentrations of MC-LR, only the high dose group (100 μg L^−1^) revealed a significant increase of GST enzyme activity in both gills and hepatopancreas of *V. philippinarum*. A possible explanation for these results may be attributed to a higher conjugation rate of MCs with GSH and/or detoxification of oxidative stress products through GST activity [15]. Furthermore, GST activity levels were consistently lower in hepatopancreas than in gills. A similar tissue specificity was shown in former reports, not only in *V. philippinarum*, but also in other bivalve species (e.g., *Perna viridis*, *Mytilus galloprovincialis*) [43,44,45]. Previously, Reis *et al.* [37] perceived a non-significant dose dependent increase of GST activity for both gills and hepatopancreas of *V. philippinarum* at 24 h post-exposure to MC-LR (10 and 100 μg L^−1^). Several other reports with bivalves also revealed an enhancement of GST activity after exposure to toxicants such as cyanotoxins combination (MC-LR+-YR), metals and polycyclic aromatic hydrocarbons (PAH) [36,43,45].

The higher basal SOD activity in the hepatopancreas of *V. philippinarum* warrants this last organ a more active role in the organism’s defense against oxidative stress at 24 h post-exposure to MC-LR. It is suggested that gills exhibit a lower response to oxidative stress since they are one of the first organs to come in contact with the contaminants [46]. This was also the case of the bivalves *V. philippinarum* exposed to a heavy metal and a PAH, and *Unio pictorum* from two river sites, where the digestive gland had a higher SOD activity than the gills upon 24 h exposure, and in the second study, the gills only had a basal level [45,46]. According to our results, the changes in GST activity following acute exposure to MC-LR can potentially result from oxidative stress in the hepatopancreas but not in the gills.

Although non-significant, there is a decrease in PO_4_ with the rise in exposure’s intensity in the gills. Martins *et al.* [28] also verified a decrease in PPP2 activity in *Corbicula fluminea* when exposed to MC-LR. On the other hand, another study exposing the mussel *Dreissena polymorpha* to MC-LR did not inhibit mRNA levels of PPP2 [47]. Nonetheless, the difference in PPP2 inhibition between both organs is noteworthy. The resulting higher PPP2 inhibition in the gills alongside the higher GST activity appear to confirm that the organism molecular response point to the MC-LR exposure.

These results suggest different MC-LR toxic pathways between both organs, with PPP2 pathway associated to gills and ROS pathway to hepatopancreas [48]. Thus, the gills reflect early-phase responses as external organs in direct contact with contaminants, whereas the hepatopancreas reflects late-phase responses, as internal tissues.

Recently, gene expression has been revealed as a useful tool to assess ecotoxicological effects of several xenobiotics [37,49,50,51,52]. Hyperphosphorylation alongside with the pro-oxidative environment inside the cell due to toxin exposure, promotes the migration of the transcriptional factor Nrf2 to the nuclei and augments Nrf2’s mean life. This occurrence promotes the transcription of genes involved in antioxidant responses, such as GSTs [53]. Although analysis of GST activity can give a broader view of GSTs response to a toxicant exposure, the real-time PCR method provides a more detailed result considering the GST enzymatic analysis. Several studies have assessed the different effects on the transcriptions of GST genes in fish and bivalves exposed to MC-LR [37,50,51,52]. Individually, GST transcriptional responses in our study revealed a significant up-regulation of sigma2 in the hepatopancreas at the high dose exposure, but no significant changes were detected in the gills. Nevertheless, for this last tissue, an increase transcription trend is perceived for pi, sigma1 and sigma2 also upon the 100 μg L^−1^ MC-LR exposure. In another work, Umasuthan *et al.* [54] injected *V. philippinarum* with bacterial lipopolysaccharide (LPS) and showed no significant changes for GST sigma gene expression in several organs, including the gills, 24 h post-injection. Interestingly, the GST gene expression was significantly elevated at 3 h post-injection, and similar extent of induced transcription was also detected at 12 h and 48 h post-injection. In contrast to gills, GST sigma transcription in hemocyte was noticed to be considerably stimulated at a later phase [54]. However, our results are consistent with those obtained previously by Reis *et al.* [37] after studying the time-dependent changes on gene expression of the same GST isoforms in *V. philippinarum* exposed to MC-LR (10 and 100 μg L^−1^). In this denoted study, MC-LR exposure caused non-significant GST transcriptional changes in the gills at 24 h post-exposure, whereas in the hepatopancreas, although non-significant, an increase trend was detected in GST sigma2 transcription reaching 3.9-fold for the high dose group [37]. Several other studies have reported identical changes in the expression of pi, mu, sigma1 and sigma2 GST isoforms with MCs or other xenobiotics [49,50,52,55,56]. Li *et al.* [52] reported a decreased of GST pi transcription after 24 h in the liver of the freshwater fish *Carassius auratus* intraperitoneally exposed to 50 μg L^−1^ of body weight of MCs extract. On the other hand, in a similar work with the same fish, Hao *et al.* [50] reported an induction of GST mu transcription after 24 h exposure to 200 μg L^−1^ of body weight of a cyanobacterial crude extract. In our experiment, the transcription of GST pi isoform decreased and GST mu isoform was induced in hepatopancreas at 24 h post-exposure, although non-significantly. Zhang *et al.* [46] evaluated the mRNA expression for several GST isoforms in *V. philippinarum* hepatopancreas exposed for 24 h to PAHs and metals. The expression of GST sigma2 transcripts were significantly up-regulated in hepatopancreas from Cu and B[α]P-exposed animals and significantly down-regulated with the exposure to Cd [49]. On the other hand, Zhang *et al.* [49] reported a decrease of GST sigma1 expression at 24 h post-exposure to B[α]P, while Cu promoted an increase of the same GST isoform levels. In what it concerns to GST sigma isoforms in hepatopancreas, our results show a significant induction of GST sigma2 transcription upon 100 μg L^−1^ MC-LR exposure, whereas GST sigma1 remained close to control levels for all MC-LR concentrations. According to the same authors, the early- and late-phase responses of GST isoforms against different challenges in external and internal tissues, respectively, account for its significant physiological role in clam post-challenges [54]. GST transcriptional changes in gills promoted by MC-LR in *V. philippinarum* were also characterized by an early (12 h) induction of mu and sigma1 transcripts and in hepatopancreas by a later induction (48 h) of mu transcript, but also by an early inhibition (6 h) of the four transcripts [37]. In our study, after 24 h of exposure, gills as an external organ probably denotes an adaptation-phase to MC-LR exposure, whereas in the hepatopancreas, the late-phase responses are taking place. Finally, our results also show differences in basal levels of tissue expression among the tested GST isoforms. These indicate that sigma2 is the most abundantly expressed in gills and in hepatopancreas, although in this no significant changes were found between pi and sigma2 genes. In the same way, Zhang *et al.* [49] similarly highlighted the importance and sensitivity of the GST isoform sigma2 in *V. philippinarum*, reporting that GST sigma2 transcripts are predominantly expressed in both tissues in relation to other several GST isoforms.

Posterior kinetic analysis of the high dose group purified cGSTs evidenced differences between exposed and non-exposed gill extracts. The rise of the apparent *V*_max_ value of the exposed gills to MC-LR might indicate an alteration in GST mixture, namely in terms of the number of different GST isoforms or relative abundance.

Using proteomics, qualitative and quantitative differences were found between the basal and inducible cGSTs mixtures. The exposed extracts presented one more putative pi-class GST subunit compared to non-exposed extracts, with upregulation of almost all putative detected GST subunits (pi and sigma2) with the exception of a pi-class GST subunit (Spot 1), which was significantly downregulated. The detection of only two different types of GST classes (pi and sigma2) could be related to an imperfect purification approach that could have not retained other isoforms and allowed the co-purification of histone along with the purified GST proteins obtained. Nevertheless, according to previous studies, the majority of GSTs expressed in bivalve mollusks seem to belong to the pi class and a small share to alpha, mu and sigma classes, which agrees with our results [21,57,58,59,60,61].

## 4. Experimental Section

### 4.1. Biological Material

*M. aeruginosa* (strain 91094, from LEGE culture collection), and *Chlorella vulgaris* (strain Z-001, from LEGE culture collection) were cultured in Z8 medium [62], using fluorescent light under a light:dark period of 14 h:10 h, and a temperature of 25 ± 1 °C. The strain of *M. aeruginosa* used in this study is a major producer of MC-LR (95%), and of small amounts of microcystin-LA (MC-LA) and [D-Asp3]-MCYST-LR [29,63].

Bivalves from the species *V. philippinarum* (average length of 4.35 ± 0.20 cm) were purchased from a local producer (ConchaMar, Foz do Arelho, Portugal). They were transported in thermal boxes to the laboratory within 24 h, on March 2014. The animals were acclimated in 5 L glass aquariums with 10 individuals per aquarium, at 16 ± 1 °C, 31.7‰ salinity, with aeration and a natural light:dark regimen. They were maintained in these conditions for 3 days prior to exposure experiments. The artificial seawater was renewed every day. Animals were fed with an algal suspension of *C. vulgaris* (10^5^ cells mL^−1^) every other day.

### 4.2. MC-LR Purification

#### 4.2.1. MC-LR Extraction

The biomass culture of *M. aeruginosa* was collected by centrifugation at 4600× g for 10 min at 4 °C. The extraction solvent (aqueous methanol—50% MeOH) was used to ressuspend the pellets that were posteriorly sonicated (VibraCell 50-sonics & Material Inc., Danbury, CT, USA) in an ice bath, at 60 Hz for 5 × 1 min to induce cell lysis. The mixtures were centrifuged again at 4600× g for 10 min at 4 °C. The supernatants were stored and the pellets underwent the same extraction protocol once more. The supernatants resulting from both steps of extraction were combined and stored at 4 °C until further use. The concentrated MC-LR extract was thereafter purified and quantified by HPLC-PDA (High-performance liquid chromatography-Photodiode Array Detector, Waters, Milford, MA, USA).

#### 4.2.2. MC-LR Purification by Semi-Preparative HPLC

The MC-LR semi-preparative assay was performed using a C18 reversed phase column (Phenomenex Luna RP-18, Torrance, CA, USA; 25 cm × 10 mm, 10 μm) kept at 45 °C. The isocratic elution was done with MeOH 60% acidified with 0.1% trifluoroacetic acid (TFA) with a flow rate of 1.5 mL/min. The injected volume ranged between 200–500 μL. Peak purity and percentage of purified MC-LR was calculated at 214 and 238 nm. Afterwards, the solution containing the purified MC-LR was dried in vacuum (Centrivap concentrator, Labconco, Kansas City, MO, USA) in order to remove the MeOH used during the extraction protocol.

#### 4.2.3. Quantification of MC-LR by HPLC-PDA

The MC-LR purified fractions were then quantified in the same HPLC system on a Merck Lichrospher RP-18 (Kenilworth, NJ, USA) endcapped column (250 mm × 4.6 mm i.d. 5 μm) equipped with a guard column (4 × 4 mm, 5 μm) both kept at 45 °C. The PDA range was 210–400 nm with a fixed wavelength of 238 nm. The linear gradient elution consisted of (A) MeOH + 0.1% TFA and (B) H_2_O + 0.1% TFA (55% A and 45% at 0 min, 65% A and 35% B at 5 min, 80% A and 20% B at 10 min, 100% A at 15 min, 55% A and 45% B at 15.1 and 20 min) with a flow rate of 0.9 mL/min. The injected volume was 20 μL. All HPLC solvents were previously filtered (Pall GH Polypro 47 mm, 0.2 μm) and degassed by ultrasound bath.

The MC-LR was identified by comparison of spectra and retention time with a standard of MC-LR (batch n° 018K1209, 10.025 μg mL^−1^ in MeOH, 98% purity, Cyano Biotech GmbH, Berlin, Germany). The system was calibrated by using a set of 7 dilutions of MC-LR standard plus another 2 sets of dilutions during the sample readings (0.5 to 20 μg mL^−1^), all in MeOH 50%. Empower 2 Chromatography Data Software (Waters, Milford, MA, USA) was used for calculation and reporting peak information. The minimum amounts of MC-LR that can be detected in water is 0.2 μg mL^−1^, based on a signal-to-noise ratio of 3. The retention time of the MC-LR peak was 8.70 min (data from method validation not published).

### 4.3. Exposure Experiment

Aquaria containing 2.5 L of artificial seawater were used for the experiments. Ten animals were placed in each aquarium. Exposure to the toxin was accomplished with a toxin cocktail that consisted of purified MC-LR mixed with *C. vulgaris* to prevent inhibitory effect by MC alone. Animals were exposed to 10, 50 and 100 μg L^−1^. The control group (0 μg L^−1^) was only fed with *C. vulgaris*. Control and treatment groups were carried out in triplicate. After 24 h, all animals from each aquarium were retrieved and pooled. Clams were weighed, measured and the organs (gills and hepatopancreas) were dissected on ice. The different organs were immediately frozen in liquid nitrogen and stored at −80 °C until further use.

### 4.4. Sample Preparation

Protein extraction began with the pulverization of the frozen tissues followed by solubilization in buffer (0.1 M phosphate buffer pH 6.5, 1.4 mM DTT, 0.1 mM EDTA, 10% v/v glycerol and Halt Protease Inhibitor Cocktail (Thermo scientific, Waltham, MA, USA). A total of 5 mL of solubilization buffer was used per gram of tissue. Samples were sonicated and subsequently centrifuged to remove cell debris at 4600× *g* for 10 min. The supernatant was ultracentrifuged at 100,000× *g* for 60 min and the post-mitochondrial extract was collected. All steps were executed at 4 °C. The supernatants of the gills samples were concentrated using vivaspin 2 mL columns (sartorius) by centrifugation at 4600× *g* for 10 min (4 °C). The previous fraction was refined with GSH-agarose prepacked columns (0.2 mL resin bed; Pierce Glutathione Spin Columns, Waltham, MA, USA), in order to purify GST-fusion proteins. All samples were stored at −20 °C until posterior use.

### 4.5. Enzymes

All enzyme activities and quantitative measurements were performed in the cytosolic fraction of both organs, the gills and the hepatopancreas, of *V. philippinarum* using a microplate reader. Protein content was previously measured according to Bradford [64].

#### 4.5.1. GST

Measurement of cytosolic GST activity was done by using the substrate 1-chloro-2,4-dinitrobenzene (CDNB) at 340 nm, according to Habig *et al.* [22] and Frasco and Guilhermino [65].

#### 4.5.2. SOD

In order to determine SOD and SOD-like activities, a commercial kit (Sigma-Aldrich, St. Louis, MO, USA) using Dojindo’s water-soluble tetrazolium salt (WST-1 (2-(4-iodophenyl)-3-(4-nitrophenyl)-5-(2,4-disulfophenyl)-2*H*-tetrazolium) was employed. The reduction of WST-1 with O_2_- is inhibited by SOD, and the rate of this reduction is linearly related to the xanthine oxidase (XO) activity. The absorbance of the reduction of WST-1 was measured at 450 nm and at 37 °C. SOD-like activity was expressed as the inhibition rate (%) of the formation of water-soluble formazan from WST-1 and a superoxide anion generated from the xanthine-xanthine oxidase system.

#### 4.5.3. PPP2

The cytosolic samples were dialyzed using Slide-A-Lyzer MINI Dialysis Device, 10K MWCO (Thermo scientific, Waltham, MA, USA) in order to exchange the solution buffer to 50 mM Tris-HCl pH 7.4 (150 mM NaCl, 5 mM DTT, 2 mM EDTA, 0,1% Triton). PPP2 activity was determined using a serine/threonine phosphatase assay kit (Promega, Madison, WI, USA) according to the manufacturer’s instructions. A synthetic phosphopeptide RRA(pT)VA, that is compatible with PPP2 was used as substrate. The samples were incubated with this substrate in the presence of a reaction buffer specific for PPP2 (250 mM imidazole, 1 mM EGTA, 0.1% β-mercaptoethanol and 0.5 mg mL^−1^ BSA) for 5 min. The reaction was stopped and the absorbance of the molybdate malachite green: phosphate complex formed in the standards, samples and respective blanks was measured after 15 min incubation at 630 nm at 25 °C.

### 4.6. Gene Expression

#### 4.6.1. RNA Extraction

Total RNA was extracted from exposed and control animals according to Qiagen’s RNeasy Mini kit protocol (Venlo, The Netherlands) for purification of total RNA from animal tissues. First, the pooled and grounded organs were transferred to a suitable vessel and 600 μL of homogenization buffer (RLT buffer) was added. Disruption and homogenization of the tissues were carried out using Precellys^®^ 24 (Bertin Technologies, Montigny le Bretonneux, France) tissue homogenizer (Bertin Technologies, Montigny le Bretonneux, France). The lysate was then centrifuged at full speed for 3 min and the supernatant was transferred to a new vessel where it was mixed with 70% ethanol (1:1). After this step, 700 μL of the sample was transferred to an RNeasy spin column, which was centrifuged for 15 s at 8000× *g* and the flow-through was discarded. Subsequently, 700 μL of RW1 buffer was added to the spin column, which was centrifuged as referred above. Afterwards, washing steps were continued by addition of 500 μL of RPE buffer with the centrifugation in the same conditions. Before elution a last washing step was performed with 500 μL of RPE buffer and the column was then centrifuged for 2 min at 8000× *g* and the flow-through discarded. Finally a total volume of 30 μL of sample was eluted using RNase-free water. A master solution containing Quant-IT reagent (Invitrogen, Carlsbad, CA, USA) (1 μL × n samples) and Quant-IT working solution (199 μL × n samples) was prepared for quantification of RNA content of the samples. Afterwards, 190 μL of master solution plus 10 μL of Quant-IT broad range RNA were mixed to prepare the standards used to quantify sample RNA: Tubes containing 1 μL of sample RNA and 199 μL of master solution were prepared, vortexed and incubated for 2 min at room temperature. Finally, RNA concentration was measured photometrically with Qubit Fluorometer (Invitrogen, Carlsbad, CA, USA).

#### 4.6.2. cDNA Synthesis

Total cDNA for the real-time polymerase chain reaction (PCR) were generated from 500 ng of total RNA from all samples according to NZYtech’s first-strand cDNA synthesis kit protocol (Lisbon, Portugal). For each reaction 10 μL of NZYRT 2× Master mix (a mixture of reaction buffer, poly dT and random primers), 2 μL of reverse transcriptase and RNA template and Nuclease-free water were used until a total volume of 20 μL. The tubes were transferred to a PCR cycler (Biometra^®^ TGRADIENT, Göttingen, Germany). The reaction conditions were as follows: 10 min at 25 °C, 30 min at 50 °C and 5 min at 85 °C. Additionally, 1 μL of RNase H (*E. coli*) was added to the mixture and the reaction vessel was incubated for 20 min at 37 °C. The enzyme was inactivated by heating at 85 °C for 5 min.

#### 4.6.3. Primers Design

All primers were obtained from Invitrogen (Carlsbard, CA, USA). Specific primers (Table 2) used for this study were taken from the following bibliography: Zhang *et al.* [43] (GST sigma 1, 2 and mu); Xu *et al.* [44] (GST pi). Specific primers were also designed for elongation factor 1-α (EF1-α) after obtaining *V. philippinarum* EF1-α sequence, using specific primers designed for the flat oyster, *Ostrea edulis* [45]. The PCR products using the specific primers were sent for sequencing to confirm the specificity of the amplified products.

**Table 2:** Primer pair sequences and product length. Genes quantified through Real-Time PCR.

GST Gene	Primer Sequence (5'-3' order)		Product Length (bp)
	Forward	Reverse	
sigma1	CAGAAGAATTTGGCAGAAGTAG	AAGACAGCAAGATCAGCGAG	121
sigma2	AAGGCTAAACTTACAGAGGAG	GTGTTTCTTGAGTTCAGGGT	209
mu	GACTTCCCAATGTACGAGCTT	ACACTTTCCTGAGCGAGATAC	139
pi	GCATTACCGACCCTCAAAGC	CCATTGACGGGCATTTTCTT	101
EF1-α	GCTCACAGAAGCTGTACCAGG	CTGGGCATAGAAGCTTGCAG	136

#### 4.6.4. Quantitative RT-PCR

Quantitative real-time PCR was performed using an iCycler iQ™ Real-Time PCR Detection System (Bio-Rad, Hercules, CA, USA). The following genes were examined in the qPCR experiments: GST pi, mu, sigma1, sigma2 coding for GST pi, mu and sigma enzymes. EF-1α was used as a control gene for DNA level normalization. The EF-1α was previously used as reference gene in other studies [29,57]. Sample cDNA was 5-fold diluted with ultra-pure H_2_O. Each reaction mixture consisted of 2 and 4 μL of cDNA template for hepatopancreas and gills, respectively, 0.25 μM of each primer; 1 × IQ SYBR Green Supermix (Bio-Rad, Hercules, CA, USA) and water to adjust to 20 μL final reaction volume. The 96-well plate was then transferred to a qPCR cycler (Biorad^®^ IQTM, Hercules, CA, USA). The qPCR conditions for *V. philippinarum* genes and reference gene, were as follows: 95 °C of initial denaturation for 30 s; 40 cycles at 95 °C for 10 s, 60 °C (GST mu, pi, sigma1 and sigma2) for 20 s and 72 °C for 20 s. A melting curve was generated for every run to confirm specificity of the assays. A cDNA pool of hepatopancreas and gills from control animals (0 μg L^−1^) was used for normalization between qPCR runs. Efficiency tests were performed to examine the quality of PCR reaction and all assays showed efficiencies between 98.9% and 110%. Efficiency is an important parameter for the calculation of gene expression; it reports fold increase between cycles. Cycle Threshold data analyses were carried out according to Pfaffl method and following Equation (1) [66]:

(1) $$R=\frac{{\left(E\text{target}\right)}^{\text{ΔCttarget(control-treated)}}}{{\left(E\text{reference}\right)}^{\Delta \text{Ctreference(control-treated)}}}$$

in which *R* = ratio, *E* = efficiency, ref = reference gene, and target = target genes. Cycle Threshold (Ct) describes the cycle number, at which the fluorescence signal gains in strength exponentially. This signal relates to PCR products amplification, in samples with increasing cDNA template, Ct will decrease.

### 4.7. Kinetic Characterization

Due to small amounts of protein, one replicate was used for each condition in the kinetic characterization assay. This analysis was employed in order to discern obvious differences among treatments and/or organs to proceed for the 2DE analyses. Kinetic analysis was performed using the purified GST fractions of both organs from organisms exposed to the highest concentration only (100 μg L^−1^) and from the non-exposed organisms. Five different concentrations of CDNB substrate were used (2, 1, 0.5, 0.25, 0.125 mM), with a fixed concentration of GSH (1 mM) and GST (0.04 mM). The assay of the enzymes at each substrate concentration was replicated three times in each plate. Absorbance was read for 5 min for every 20 s at 340 nm at 25 °C. Data were fitted to the Michaelis-Menten model in order to determine the Michaelis-Menten constant (Km) and maximal velocity (*V*_max_). *V*_max_ and *K*_m_ values were determined using nonlinear regression.

### 4.8. Two-Dimensional Gel Electrophoresis (2DE)

#### 4.8.1. Isoelectric Focusing

The 2DE analysis was performed on the gills of the 100 μg L^−1^ and the control groups according to the results of the kinetic characterization. The procedure was based on the one described by Campos *et al.* [67]. Each sample was leveled to 29.4 μg protein and was diluted in rehydration buffer (7 M urea, 2 M thiourea, 0.8% *v*/*v* immobilized pH gradient (IPG) buffer, 4% *w*/*v* 3-[(3-Cholamidopropyl)-dimethylammonio]-1-propane sulfonate (CHAPS), 65 mM dithiothreitol) with bromophenol blue to a final volume of 125 μL. Samples were thereafter loaded in IEF gel strips with 7 cm length, and linear pH gradient 4–7 IPG strip (Bio-Rad, Hercules, CA, USA). Afterwards, the Protean IEF Cell (Bio-Rad, Hercules, CA, USA) separated the proteins by isoelectric focusing performed at 20 °C with the following program: 50 V for 12 h (strip rehydration), 15 min at 250 V, 2 h voltage gradient to 4000 V (linear ramp) and accumulated voltage to 20,000 V/h (linear ramp). Once the IEF was over, the strips were transferred to a strip holder tray and stored at −20 °C until the second dimension electrophoresis run.

#### 4.8.2. SDS-PAGE

The IEF gel strips were equilibrated in two steps according to Görg *et al.* [68]. The first was performed with equilibration buffer (50 mM Tris-HCl pH 8.8, 6 M urea, 30% *v*/*v* glycerol, 2% *w*/*v* SDS, and traces of bromophenol blue) plus dithiothreitol (DTT) (10 mM), and the second with equilibration buffer to which iodoacetamide (25 mM) was added. Protein markers were prepared by adding pre-stained SDS-PAGE standards (Bio-Rad, Hercules, CA, USA), and placed, along with the IEF gel strips above the resolving SDS-PAGE gel (12% w/v acrylamide, 0.375 M Tris-HCl pH 8.8, 0.1% SDS, distilled H_2_O, 0.05% ammonium persulfate and 0.05% Tetramethylethylenediamine). We secured the IEF gel strip and the marker with an agarose solution (0.5% *w*/*v*). The casting chamber was then filled with running buffer (25 mM Tris, 192 mM glycerine, 0.1% SDS). Proteins were subsequently transferred and separated in a SDS-PAGE gel, using a mini Protean Cell (Bio-Rad, Hercules, CA, USA), at 30 V for 30 min, and then 150 V until the bromophenol dye front reached the end of the gel. After electrophoresis, the gels were taken out of the glass plates and stained with colloidal coomassie blue [69].

#### 4.8.3. Image Acquisition and Analysis

2DE Gel images were acquired with the GS-800 Calibrated Densitometer (Bio-Rad, Hercules, CA, USA) and with the Quantity One software (Bio-Rad, Hercules, CA, USA). Protein spots were detected automatically with the PDQuest 2-D Analysis Software (Bio-Rad, Hercules, CA, USA). Protein spot intensities were normalized regarding the total density in the gel image. This allowed for a better comparison of spot quantities between gels. A synthetic image containing the spot data from all the gels, the master gel, was created. After optimizing the image display and filtering, spot matching was performed by the software and was followed by manual inspection of the matches. Differences in protein expression between experimental groups were assessed by performing qualitative and quantitative analyses. Only spots present in 2 out of 3 gels of either treatment were considered for subsequent analysis. Spots corresponding to differentially expressed proteins were considered only if they had a minimum 4-fold difference over background density and a minimum 4-fold difference relative to control and/or a significance level of *p* < 0.10 (Student’s *t*-test). Spots that matched all criteria were selected for peptide mass fingerprinting analysis.

#### 4.8.4. In-Gel Digestion of Proteins

Spots of interest were manually excised from the gels, placed in sterile microcentrifuge tubes containing ultrapure water and stored at 4 °C until digestion. Gel plugs were destained by a first wash of 50 mM ammonium bicarbonate (ABC)/5% acetonitrile (ACN) and two additional washes of 50 mM ABC/50% ACN. The gel plugs were dried with 100% ACN, which were then rehydrated with a solution of trypsin (sequencing grade; Promega, Madison, WI, USA) (10 mg mL^−1^). Digestion occurred overnight at 37 °C.

#### 4.8.5. MALDI-TOF/TOF and MS/MS Analysis and Database Search

Peptide extraction was performed by a 60% ACN/0.1% TFA solution (twice). Protein digests were desalted and concentrated using ZipTips (Millipore, Bedford, MA, USA) following the manufacturer’s instructions. Samples were crystallized onto a stainless steel 192-well MALDI plate using the dried droplet method. For the matrix, a solution of 5–10 mg mL^−1^ a-cyano-4-hydroxycinnamic acid in 50% ACN/0.1% trifluoroacetic acid was used. Samples were analyzed using a 4800 Plus MALDI TOF/TOF Analyzer (AB SCIEX, Framingham, MA, USA) as described [70]. Peptide mass fingerprint (PMF) data was collected in positive MS reflector mode within the ion range at *m*/*z* 700–4000 and was calibrated internally using trypsin autolysis peaks. Twenty-five of the highest intensity and/or relevant non-tryptic peaks were selected for MS/MS analysis. Both MS and MS/MS spectra were analyzed using the Mascot [71] search engine software (version 2.5) (Matrix Science, London, UK). The UniProt protein sequence database (release 2014_09) was considered for this analysis using the taxonomic classification *V. philippinarum*. The search included peaks with a signal-to-noise ratio greater than 20 and allowed for up to two missed trypsin cleavage sites. To be considered a match, a protein score greater than 37 was required (*p* < 0.05).

### 4.9. Statistical Analysis

The results are depicted as mean ± standard deviation (SD). Parametric tests were used to seek significant differences between means. A part from the 2DE analysis, these tests included one-way analysis of variance (ANOVA) and the Tukey test. Outliers, when found, were removed according to the Bonferroni outlier test. Only results with *p* < 0.05 were considered statistically different.

## 5. Conclusions

MC-LR promoted changes in activity and transcriptional GST responses on both gills and hepatopancreas of *V. philippinarum*. A significant increase in GST activity has been shown for both organs upon exposure to the high MC-LR dose group. Interestingly, this alteration seems to result from different MC-LR toxic pathways in each tissue (ROS and PPP2 pathways). Transcription responses revealed a significant up-regulation of sigma2 in hepatopancreas at the high dose group, but no significant changes in gills. Proteomics revealed qualitative and quantitative differences in the induced gills cGST mixtures. Overall, the results of the present study suggest a distinct role of GST system in counteracting MCs toxicity between gills and hepatopancreas of *V. philippinarum*. Transcriptional and proteomic results also evidence individual response differences among GST isoforms to MCs, within and between both organs.
